# Perceived age discrimination in older adults

**DOI:** 10.1093/ageing/aft146

**Published:** 2013-09-26

**Authors:** Isla Rippon, Dylan Kneale, Cesar de Oliveira, Panayotes Demakakos, Andrew Steptoe

**Affiliations:** 1 Department of Epidemiology & Public Health, University College London, London, UK; 2 International Longevity Centre – UK, London, UK

**Keywords:** discrimination, ageing, ageism, ELSA, England, older adults

## Abstract

**Objectives:** to examine perceived age discrimination in a large representative sample of older adults in England.

**Methods:** this cross-sectional study of over 7,500 individuals used data from the fifth wave of the English Longitudinal Study of Ageing (ELSA), a longitudinal cohort study of men and women aged 52 years and older in England. Wave 5 asked respondents about the frequency of five everyday discriminatory situations. Participants who attributed any experiences of discrimination to their age were treated as cases of perceived age discrimination. Multivariable logistic regression analysis was used to estimate the odds ratios of experiencing perceived age discrimination in relation to selected sociodemographic factors.

**Results:** approximately a third (33.3%) of all respondents experienced age discrimination, rising to 36.8% in those aged 65 and over. Perceived age discrimination was associated with older age, higher education, lower levels of household wealth and being retired or not in employment. The correlates of age discrimination across the five discriminatory situations were similar.

**Conclusion:** understanding age discrimination is vital if we are to develop appropriate policies and to target future interventions effectively. These findings highlight the scale of the challenge of age discrimination for older adults in England and illustrate that those groups are particularly vulnerable to this form of discrimination.

## Introduction

The population in England and other countries continues to age due to a decrease in fertility coupled with an increased life expectancy. With people living longer, age discrimination is likely to gain greater prominence, which in turn has important implications for social protection, access to health and other public services, and securing the human rights of the older population. A key aspect that separates age discrimination from other forms of discrimination or unfair treatment is that everyone is potentially at risk of experiencing it at some point in their lives [1]. The term age discrimination is often used interchangeably with the term ageism. The term ageism was first introduced by Butler in 1969, who defined it as: ‘a systematic stereotyping of and discrimination against people because they are old, just as racism and sexism accomplish this with skin color and gender’ [2].

The extent of the problem in the UK is difficult to establish, since high-quality evidence from large-scale representative population surveys of older people is limited. Questions about age discrimination have been included in Eurobarometer surveys [3, 4], but the samples in each country have included relatively few older people. Items about age discrimination were included in the 2008 European Social Survey, and showed wide variations in the prevalence of discrimination across countries [5, 6]. However, on average 26% of respondents aged 62 and older said they sometimes and 11% that they frequently experienced discrimination on account of their age [5]. Another study involving 1,301 people aged 50 and over found that 23% of respondents had experienced age discrimination in the past year [7]. In order to enhance current knowledge, the first objective of our study was to examine the extent of perceived age discrimination in a large nationally representative sample of >7,500 men and women aged 52 and older, assessed as part of the English Longitudinal Study of Ageing [8].

Discrimination is thought to have a profound effect on the wellbeing of the individual [5, 9]. Perceptions of discrimination have been related to heightened physiological and psychological stress responses [10]. It has been argued that as a chronic stressor, perceived day-to-day discrimination can build up over time and eventually affect both an individual's physical and mental health [11, 12]. Frequent exposure to perceived age discrimination could lead to social withdrawal, reduction in cultural engagement and reluctance to visit health professionals.

The majority of previous research—predominantly from the USA—has focussed on experiences of perceived discrimination in general and its association with race and ethnicity [13–17]. Those studies that have considered perceived age discrimination indicate that besides age, experiences of age discrimination have variously been found to be associated with gender, lack of paid employment, not being married, ethnicity, years of education and lower socioeconomic status (SES) as defined by household income or occupational social class [5, 7, 18–21]. However, to our knowledge, very few studies have looked specifically at the correlates of perceived age discrimination in older age groups in the UK. Therefore, the second objective of our study was to explore the sociodemographic factors that are related to experiences of perceived age discrimination in everyday situations.

## Methods

Data were drawn from Wave 5 (2010–11) of the English Longitudinal Study of Ageing (ELSA). ELSA is a longitudinal panel survey of ageing and quality of life among men and women aged 50 and older living in private households in England, which commenced in 2002–03 [8]. The sample is reassessed every 2 years, and is periodically refreshed to ensure a representation of younger participants. Among the 9,090 core participants who were interviewed at Wave 5 of ELSA, 8,107 (93% of those eligible) answered the self-completion questionnaire that contained the measures of age discrimination. After exclusion of 302 (3.7%) participants due to missing data, our analytical sample comprised 7,805 respondents.

### Measures

Wave 5 of ELSA was the first to include questions on experiences of discrimination. These were based on the items developed and used widely in other longitudinal studies in the USA, notably the Health and Retirement Survey (HRS) and the Midlife in the United States (MIDUS) survey [9, 11, 16, 18, 22, 23].

#### Perceived discrimination

Respondents were asked about the frequency of five discriminatory situations as follows: ‘In your day-to-day life, how often have any of the following things happened to you?’

- You are treated with less respect or courtesy
- You receive poorer service than other people in restaurants and stores
- People act as if they think you are not clever
- You are threatened or harassed
- You receive poorer service or treatment than other people from doctors or hospitals

Possible response options ranged from 1 (almost every day) to 6 (never). The responses were dichotomised to indicate whether or not participants had experienced discrimination in the past year (a few times or more a year vs. less than once a year or never), with the exception of the fifth item which was dichotomised to indicate whether or not respondents had ever experienced discrimination from doctors or hospitals (never vs. all other options). A follow-up question asked respondents to indicate what reason/s they attributed their experience to in any of the five discriminatory situations. Possible options included: age, gender, race, weight and physical disability, and participants were able to select more than one reason. Participants who attributed any experiences of discrimination to their age are treated in our study as cases of perceived age discrimination.

#### Covariates

We included age, sex, wealth, education, marital status and current employment status as covariates. Age was split into four categories: 52–59 years, 60–69 years, 70–79 years and a final group combining all those aged 80 and over. Wealth is regarded as the best indicator of socioeconomic resources in ELSA, and was measured at the household level. It was divided into quintiles for the purpose of analysis. Education was measured by the highest educational qualification attained and divided into three groups: low (no educational qualifications), intermediate (Certificate of Secondary Education or equivalent) and high (A Levels or equivalent through to higher degrees). We coded marital status into four categories: single, married or remarried, separated or divorced and widowed. Current work status indicated whether or not a respondent was currently employed, retired or in another situation, for example, unemployed or looking after the home or family.

### Statistical analysis

All analyses were conducted using STATA 12. The primary outcome was the perception of age discrimination in any of the five discriminatory situations. The secondary outcomes were perceptions of age discrimination in each of the five situations. We used *χ*^2^ tests to assess the relationship between perceived age discrimination and individual covariates, and multivariable logistic regression analysis to estimate the odds ratios of experiencing perceived age discrimination adjusting for all covariates. The discriminatory situations were analysed in separate models to identify the significance of different sociodemographic characteristics. Interactions between age and wealth were also tested. A cross-sectional design weight was applied to all analyses to correct for non-response.

## Results

We found that approximately a third (33.3%) of all respondents experienced age discrimination, rising to 36.8% in the aged 65 and over. The descriptive analyses indicated that all the sociodemographic factors with the exception of marital status were related to perceived age discrimination. Overall, perceived age discrimination was more common in male, older, less wealthy, more educated and retired respondents. The multivariable analyses showed that perceived age discrimination increased with age, peaking in the 70–79 age group (OR 1.42; 95% CI 1.18–1.71), but that the sex difference was no longer significant (Table 1). Those with intermediate and high education were more likely to report age discrimination than those with a low level of education. In contrast, respondents in the highest wealth quintile were 35% less likely to experience perceived age discrimination in comparison with those in the lowest wealth quintile (OR 0.65; 95% CI 0.54–0.78; *P* < 0.001). The results also indicated that current work status was an important correlate of age discrimination. Employed respondents were shown to be 25% less likely to report age discrimination in comparison with those who were retired.

**Table 1.** Associations between age discrimination and sociodemographic factors

	*N* (Unweighted)	Age discrimination (%)	Unadjusted odds ratio (95% CI)	*P*-value^a^	Adjusted odds ratio (95% CI)	*P*-value^b^
All	7,805	33.3				
Over 65s	4,298	36.8				
Age in years (*n* = 7,805)						
52–59	1,717	26.6	1.00	<0.001	1.00	
60–69	3,161	35.2	1.53 (1.35–1.74)		1.36 (1.17–1.59)	<0.001
70–79	2,104	37.2	1.68 (1.46–1.93)		1.42 (1.18–1.71)	<0.001
Over 80	823	35.0	1.50 (1.25–1.79)		1.28 (1.02–1.62)	0.036
Sex (*n* = 7,805)						
Male	3,481	34.5	1.00	0.050	1.00	
Female	4,324	32.3	0.91 (0.83–1.00)		0.90 (0.78–1.10)	0.064
Wealth (*n* = 7,656)						
Lowest 1	1,243	35.8	1.00	0.018	1.00	
2	1,514	34.7	0.99 (0.85–1.15)		0.92 (0.78–1.10)	0.365
3	1,552	33.7	0.92 (0.79–1.08)		0.83 (0.70–0.98)	0.032
4	1,641	33.2	0.89 (0.76–1.04)		0.78 (0.66–0.93)	0.007
Highest 5	1,706	30.0	0.80 (0.68–0.93)		0.65 (0.54–0.78)	<0.001
Education (*n* = 7,803)						
Low	1,897	31.1	1.00	0.008	1.00	
Intermediate	2,416	33.7	1.14 (1.01–1.30)		1.26 (1.10–1.45)	0.001
High	3,490	34.5	1.21 (1.07–1.36)		1.50 (1.30–1.73)	<0.001
Marital status (*n* = 7,804)						
Single	460	32.2	1.00	0.801	1.00	
Married	5,195	33.0	1.02 (0.83–1.24)		1.07 (0.87–1.34)	0.535
Divorced or separated	935	33.4	1.07 (0.85–1.36)		1.06 (0.82–1.37)	0.674
Widowed	1,214	35.4	1.07 (0.85–1.34)		1.08 (0.83–1.39)	0.576
Work status (*n* = 7,804)						
Retired	4,661	36.8	1.00	<0.001	1.00	
Employed	2,259	28.2	0.67 (0.61–0.75)		0.75 (0.65–0.86)	<0.001
Other	884	30.8	0.75 (0.65–0.88)		0.85 (0.71–1.01)	0.059

All analyses based on weighted data. Cross-sectional survey weights for non-response were used to ensure that our results reflect the population the sample was selected from.

CI, confidence interval.

^a^*χ*^2^-test of association.

^b^Multivariable odds ratios and *P*-value are adjusted for all covariates.

Our analyses of the individual discriminatory situations indicated that the proportion of respondents who experienced age discrimination in each situation ranged from 17.7%, for those who were treated with less courtesy or respect, to 4.6%, for those who experienced harassment (Table 2). The results of multivariable analysis (Table 3) indicate that sex, wealth and level of education were the most consistent correlates in all five situations. As observed overall, age discrimination was more common among better-educated respondents, while wealth was inversely associated with discrimination. Men reported more age discrimination than women in all five situations. We found that the likelihood of attributing a discriminatory situation to age discrimination generally declined with age, with the exception of medical settings. Here the likelihood of reporting age discrimination increased in the 60–69 age group before remaining at a constant level. Retired respondents report more discrimination than those in employment although this was not statistically significant for individual situations.

**Table 2.** Percentage of sample reporting age discrimination in different discriminatory situations (*N* = 7805)

	Less courtesy		Medical setting		Harassed		Service setting		Less clever	
	*N*	%	*N*	%	*N*	%	*N*	%	*N*	%
Age discrimination	1,396	17.7	797	9.9	345	4.6	681	8.7	861	11.1
Age in years										
52–59	298	17.3	158	9.0	93	5.5	156	9.1	177	10.3
60–69	624	20.0	343	10.6	141	4.8	280	9.0	335	11.0
70–79	363	17.0	217	9.8	87	4.1	190	8.7	250	11.8
Over 80	111	13.4	79	10.0	24	2.7	55	6.3	99	11.9
Sex										
Male	720	20.7	373	10.6	205	6.2	350	10.0	397	11.9
Female	676	15.2	424	9.3	140	3.2	331	7.5	464	10.5
Wealth										
Lowest 1	264	21.0	151	11.8	71	6.0	123	10.3	197	16.3
2	293	19.1	148	9.1	86	5.7	147	9.3	203	13.0
3	296	18.6	160	10.2	52	3.4	150	9.2	166	10.4
4	295	17.9	165	9.7	78	4.9	143	8.7	161	10.0
Highest 5	228	13.0	163	9.1	54	3.2	108	6.1	122	7.0
Education										
Low	306	16.1	168	8.7	75	4.0	161	8.2	234	12.3
Intermediate	456	18.8	230	9.3	101	4.4	217	9.0	274	11.1
High	634	18.1	399	11.2	169	5.1	303	8.7	353	10.4
Marital status										
Single	84	18.2	49	10.5	32	7.0	42	9.0	52	11.4
Married	922	17.8	509	9.4	226	4.6	458	8.9	517	10.2
Divorced or separated	197	20.4	117	11.9	45	4.9	91	9.3	138	14.3
Widowed	193	15.5	122	10.4	42	3.2	90	7.1	154	12.6
Work status										
Retired	837	17.8	503	10.4	189	4.0	402	8.4	546	11.8
Employed	418	18.3	197	8.5	112	5.1	198	8.8	224	10.0
Other	141	16.4	97	11.1	44	5.7	81	9.6	91	10.8

All percentages are weighted.

**Table 3.** Adjusted odds ratios (95% CIs) from logistic regression of reporting discrimination in different discriminatory situations and attributing it to age, with sociodemographic factors

	Less courtesy		Medical setting		Harassed		Service setting		Less clever	
	Odds ratio (95% CI)	*P*-value	Odds ratio (95% CI)	*P*-value	Odds ratio (95% CI)	*P*-value	Odds ratio (95% CI)	*P*-value	Odds ratio (95% CI)	*P*-value
Age (years)										
52–59	1.00		1.00		1.00		1.00		1.00	
60–69	1.16 (0.97–1.40)	0.109	1.11 (1.24–1.72)	0.371	0.93 (0.67–1.30)	0.664	0.97 (0.77–1.25)	0.868	1.01 (0.79–1.28)	0.954
70–79	0.91 (0.73–1.15)	0.438	0.98 (1.31–1.94)	0.912	0.81 (0.53–1.23)	0.319	0.91 (0.68–1.23)	0.543	0.98 (0.74–1.30)	0.893
Over 80	0.69 (0.51–0.93)	0.016	0.98 (1.12–1.81)	0.923	0.54 (0.29–0.98)	0.042	0.66 (0.44–1.00)	0.050	0.93 (0.65–1.33)	0.688
Sex										
Male	1.00		1.00		1.00		1.00		1.00	
Female	0.68 (0.60–0.78)	<0.001	0.84 (0.72–0.99)	0.043	0.50 (0.39–0.64)	<0.001	0.72 (0.60–0.86)	<0.001	0. 81 (0.69–0.95)	0.011
Wealth										
Lowest 1	1.00		1.00		1.00		1.00		1.00	
2	0.84 (0.69–1.03)	0.078	0.75 (0.58–0.98)	0.032	0.91 (0.64–1.28)	0.579	0.85 (0.66–1.12)	0.267	0.79 (0.63–0.99)	0.042
3	0.80 (0.65–0.98)	0.028	0.83 (0.64–1.08)	0.164	0.52 (0.35–0.77)	0.001	0.84 (0.63–1.10)	0.204	0.61 (0.48–0.78)	<0.001
4	0.73 (0.59–0.90)	0.003	0.75 (0.57–0.98)	0.034	0.71 (0.48–1.03)	0.076	0.76 (0.57–1.01)	0.058	0.57 (0.44–0.74)	<0.001
Highest 5	0.47 (0.38–0.60)	<0.001	0.67 (0.51–0.88)	0.004	0.42 (0.28–0.65)	<0.001	0.50 (0.36–0.68)	<0.001	0. 38 (0.29–0.51)	<0.001
Education										
Low	1.00		1.00		1.00		1.00		1.00	
Intermediate	1.23 (1.04–1.47)	0.017	1.17 (0.93–1.46)	0.178	1.16 (0.83–1.61)	0.380	1.13 (0.89–1.42)	0.313	1.01 (0.82–1.23)	0.944
High	1.24 (1.04–1.48)	0.018	1.55 (1.23–1.95)	<0.001	1.33 (0.95–1.86)	0.094	1.14 (0.90–1.45)	0.278	1.08 (0.88–1.34)	0.456
Marital status										
Single	1.00		1.00		1.00		1.00		1.00	
Married	1.09 (0.83–1.43)	0.544	0.96 (0.69–1.34)	0.812	0.78 (0.51–1.19)	0.253	1.13 (0.79–1.64)	0.490	1.06 (0.76–1.49)	0.720
Divorced or separated	1.16 (0.85–1.59)	0.338	1.17 (0.80–1.70)	0.423	0.73 (0.44–1.21)	0.227	1.08 (0.72–1.64)	0.707	1.29 (0.89–1.86)	0.182
Widowed	1.09 (0.79–1.49)	0.597	1.10 (0.74–1.61)	0.634	0.73 (0.43–1.27)	0.274	1.01 (0.65–1.57)	0.955	1.19 (0.81–1.75)	0.380
Work status										
Retired	1.00		1.00		1.00		1.00		1.00	
Employed	0.90 (0.76–1.07)	0.107	0.75 (0.60–0.94)	0.013	0.96 (0.68–1.35)	0.817	0.89 (0.71–1.13)	0.353	0.82 (0.65–1.03)	0.099
Other	0.83 (0.67–1.03)	0.097	1.03 (0.79–1.34)	0.838	1.18 (0.80–1.75)	0.410	1.04 (0.78–1.37)	0.796	0.82 (0.62–1.06)	0.124

All analyses based on weighted data. Cross-sectional survey weights were used to ensure that our results reflect the population the sample was selected from.

Odds ratios are adjusted for the individual discriminatory scenario, age, gender, wealth, education, marital status and work status.

CI, confidence interval.

## Discussion

Our analyses indicated that approximately a third (33.3%) of this national cohort of men and women aged 52 and older experienced age discrimination, with rates increasing to 36.8% among respondents aged 65 and over. We found that perceived age discrimination was associated with older age, and was also associated with higher levels of education, lower levels of household wealth and lack of paid employment. Of the five individual discriminatory situations measured, perceived age discrimination was more prevalent where people were treated with less courtesy (17.7%) and least where people experienced harassment (4.6%). The analysis of the individual discriminatory situations revealed many similar associations, with level of education, and wealth being the most significant correlates regardless of the discriminatory situation itself.

The level of perceived age discrimination reported here is comparable with a number of previous studies outside the UK. An analysis of European Union countries found that 26% of people aged 62 and over had frequently or sometimes experienced age discrimination [5]. Studies using data from the HRS and MIDUS surveys in the US reported that ∼30% of respondents age 50 and over cited age as the most common reason for perceived everyday discrimination [9, 11, 22]. In accordance with previous studies, we found that overall perceived age discrimination increased with age [9, 11, 22].

This study revealed somewhat counterintuitive results for the relationship between perceived age discrimination and the two indicators of SES, wealth and education. In common with previous studies, we observed a negative gradient between perceived age discrimination and SES, with individuals in the lowest wealth quintile more likely to experience age discrimination in comparison with wealthier respondents [5, 7, 11, 18, 22]. In contrast, perceived age discrimination was more likely to be reported by respondents with a high level of education than those with an intermediate or low level of education. Our findings for both wealth and education are supported by some but not all previous studies on perceived discrimination [11, 18, 22]. They are consistent to overall findings from the European Union, which indicated that older adults with a higher level of education and low-household income reported more age discrimination [5, 24]. A US study of 25–74 year olds also found that respondents who were better educated and less affluent were more likely to report age discrimination than those who experienced no discrimination or discrimination due to another reason [18]. This may be due to the fact that better educated older adults more readily perceive inequities and are therefore more likely to report discrimination [22], whereas it could be argued that wealth potentially protects individuals from exposure to situations that give rise to discrimination or provides a greater sense of control or security. Further analysis of the relationship between indicators of SES and perceived age discrimination may help to clarify these observed disparities.

Our findings also indicate that respondents who were retired reported perceived age discrimination more than those who were employed. This is consistent with analysis of data from the UK, which found that working status was a strong correlate of age discrimination, with a larger proportion of respondents who were not working or were retired reporting age discrimination in comparison with those employed full time [25]. As suggested by Abrams *et al.,* this could be a result of individuals in employment perceiving old age to begin later in comparison with individuals who are retired or not working for other reasons [25]. Contrary to previous studies, we found no association between marital status and age discrimination, whereas others have reported that unmarried and separated/divorced or widowed respondents experienced more age discrimination than married people [18, 19, 25]. Our finding could suggest that an individual's identification with other sociodemographic characteristics explains their perception of age discrimination to a greater extent than marital status.

Our analyses of the individual discriminatory situations revealed rather low rates of actual harassment, which could suggest that older people are regarded as less of a target by younger generations. Eleven per cent of respondents reported being thought of as less clever because of their age. This might reflect the negative old age stereotype in which older people are regarded as incompetent. The findings from these two discriminatory situations could be seen as reinforcing the persistence of the old age stereotype where older people are regarded as both warm and incompetent [26]. Our study found that ∼10% of the whole sample reported perceived age discrimination in a hospital or from a doctor, providing further evidence of the existence of ageism in medical settings, an area that previous research has identified as a particular problem [27–29]. Age discrimination may be evident in how clinical staff communicate with older patients and in the quality of care older patients receive in comparison with younger patients [27].

Caution is needed when interpreting these findings. First, it is not possible to establish causal relationships in this cross-sectional study. We do not know whether older people are more likely to experience discrimination because of their age or whether they are more likely to attribute discrimination to age as they get older. Second, the measures of discrimination used were self-reported and therefore subject to recall bias. Third, the questions were designed to measure age discrimination in the context of other sources of discrimination, and therefore may not be optimal. However, a more targeted measure may prime respondents to answer in a particular way, whereas in our study, age was not the apparent focus of the items. Finally, respondents were able to attribute more than one reason to their experiences of discrimination; therefore, it is not possible to establish for certain whether an individual situation was due to age discrimination or another type of discrimination.

Nevertheless, what this study has been able to show though is that age discrimination is encountered in the day-to-day lives of many older adults in the UK and that it is an area that needs to be studied further in order to improve our understanding of the mechanisms through which it impacts upon the individual and society. The fact that age discrimination has been shown to affect a high proportion of individuals in later life is relevant to public policy. Understanding age discrimination is important if we are to develop appropriate policies and to target interventions effectively.

## Ethical approval

Approval for all the ELSA waves was granted from the National Research and Ethics Service (NRES).

**Key points**

- There is limited evidence from large-scale representative population surveys of older people on the extent of age discrimination in England.
- It is important to understand the potential scale of age discrimination, as it has a profound effect on the wellbeing of the individual.
- Frequent or occasional age discrimination is experienced by approximately a third of older adults in England.
- Sociodemographic characteristics associated with perceived age discrimination are older age, lower household wealth, higher levels of education and being retired or not working compared with being employed.

## Conflicts of interest

None declared.

## Funding

The English Longitudinal Study of Ageing was developed by a team of researchers based at the University College London, National Centre for Social Research and the Institute for Fiscal Studies. The data were collected by the National Centre for Social Research. The funding is provided by the National Institute of Aging in the United States, and a Consortium of UK Government Departments Coordinated by the Office for National Statistics. The developers and funders of the English Longitudinal Study of Ageing and the UK Data Archive do not bear any responsibility for the analyses or interpretations presented here. I.R. is supported by an Impact PhD studentship from the International Longevity Centre-UK (ILC-UK) and University College London. A.S. is funded by the British Heart Foundation.
